# Case of laparoscopic parastomal hernia repair using modified Sugarbaker mesh method

**DOI:** 10.1093/jscr/rjac488

**Published:** 2022-11-19

**Authors:** Yuki Tsuchiya, Hiromitsu Takahashi, Yosiro Ishibiki, Yoshimi Iwanuma, Kazuhiro Sakamoto, Masaki Fukunaga

**Affiliations:** Department of Surgery, Gastrointestinal Surgery, Juntendo Tokyo koto Geriatric Medical Center, Tokyo, Japan; Department of Coloproctological Surgery, Juntendo University Faculty of Medicine, Tokyo, Japan; Department of Surgery, Gastrointestinal Surgery, Juntendo Tokyo koto Geriatric Medical Center, Tokyo, Japan; Department of Surgery, Gastrointestinal Surgery, Juntendo Tokyo koto Geriatric Medical Center, Tokyo, Japan; Department of Surgery, Gastrointestinal Surgery, Juntendo Tokyo koto Geriatric Medical Center, Tokyo, Japan; Department of Coloproctological Surgery, Juntendo University Faculty of Medicine, Tokyo, Japan; Department of Surgery, Gastrointestinal Surgery, Juntendo Tokyo koto Geriatric Medical Center, Tokyo, Japan

**Keywords:** parastomal hernia, Sugarbaker approach, laparoscopy

## Abstract

A 75-year-old woman underwent sigmoid colon resection and transverse colostomy for perforation of the diverticulum of the sigmoid colon at 70 years of age at another hospital. She was referred to our hospital with complaints of abdominal discomfort 3 months prior to presentation. Abdominal computed tomography revealed a parastomal hernia (PSH). We performed laparoscopic repair using the Sugarbaker approach with a Symbotex Composite Mesh™ and laparoscopic adhesive intestinal repair. The patient’s post-operative course was unremarkable, and she was transferred to the Department of Internal Medicine after 10 days. There was no recurrence 6 months after surgery. Tension-free surgery using a mesh has been reported to be effective in preventing the recurrence of PSH. We performed a laparoscopic modified Sugarbaker mesh method using the Symbotex Composite Mesh™ with collagen film to repair an abdominal hernia.

## INTRODUCTION

A parastomal hernia (PSH) is a common complication of stoma formation. The incidence varies significantly and may be as high as 48% for colostomies and 28% for ileostomies [1]. Fortunately, most cases of PSH can be managed conservatively; nevertheless, they may cause annoying symptoms such as pain, leakage of stomal contents and cosmetic disfigurement. Many operative techniques have been proposed to correct symptomatic PSH, but so far, none have been able to provide satisfactory results, especially in the long term [2]. The incidence of colostomies ranges from 3 to 39%, whereas for loop ileostomy, the incidence is reported to be between 0 and 6% [1]. Most PSHs develop in the first few years after stoma formation [3].

Indications for surgery include ill-fitting appliances that cause leakage, pain, discomfort and cosmetic complaints. Surgical repair of PSH presents a challenging problem with high complication and recurrence rates. Multiple repair techniques have been described in the past few decades, but none has been established as optimal. Pure fascia repair or stoma relocation has been abandoned owing to poor outcomes. Currently, the consensus is that a nonslit-mesh-based laparoscopic repair technique is the treatment principle for this disease [4].

Various open and laparoscopic techniques have been described. However, laparoscopic PSH repair using a modified Sugarbaker technique is preferred to a keyhole mesh [4]. Here, we present a case of PSH for which we performed laparoscopic PSH repair using the modified Sugarbaker mesh method.

## CASE REPORT

**Figure 1:**
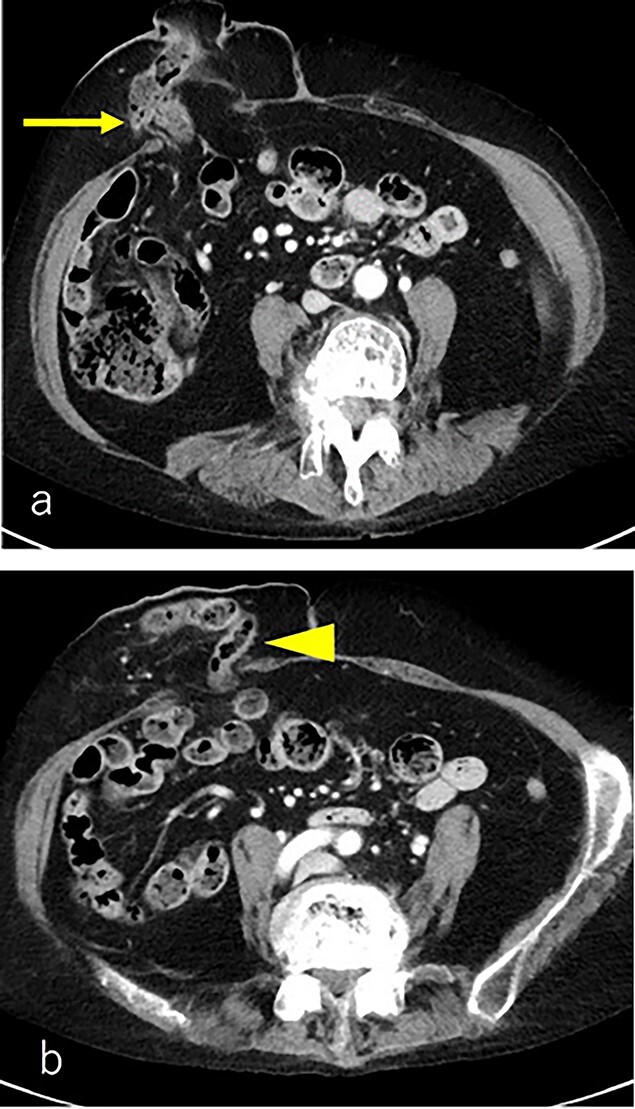
(**a**) CT scan revealed a prolapse of the transverse colon on the oral side (arrow); (**b**) CT scan revealed a prolapse of the transverse colon on the anal side (triangle).

A 75-year-old woman underwent sigmoid colon resection and transverse colostomy for perforation of the diverticulum of the sigmoid colon at 70 years of age at another hospital. The colostomy was performed in the right upper quadrant. She was referred to our hospital with complaints of abdominal discomfort 3 months prior to presentation. She had a history of rheumatoid arthritis and took steroids. Abdominal computed tomography (CT) revealed PSH with loss of fascia around her ostomy. The defect measured ~65 × 60 mm. Herniation of the transverse colon was observed (Fig. 1). No abdominal wall hernia was found, and the diagnosis was European Hernia Society (EHS) type III.

**Figure 2:**
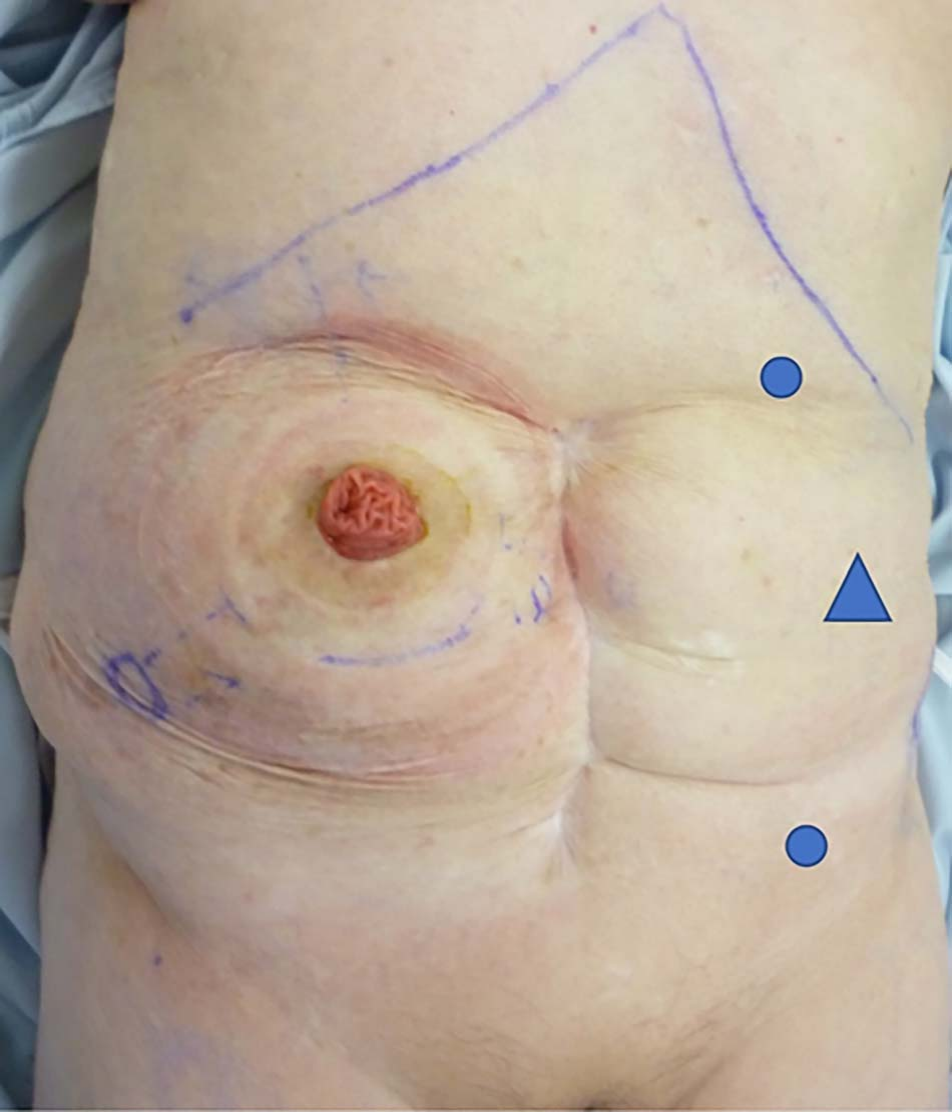
Port replacement: two 5-mm ports (circle) and 12 mm port (triangle) placed vertically in the left abdominal quadrants.

**Figure 3:**
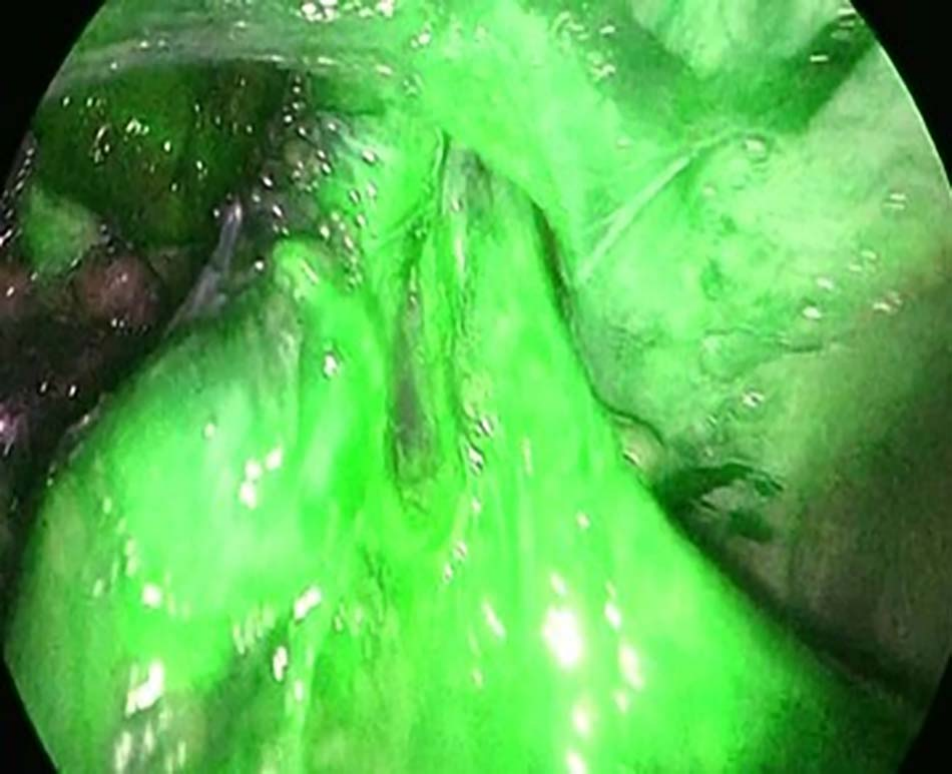
The hernia orifice was exposed and intestinal blood flow was evaluated by indocyanine green.

**Figure 4:**
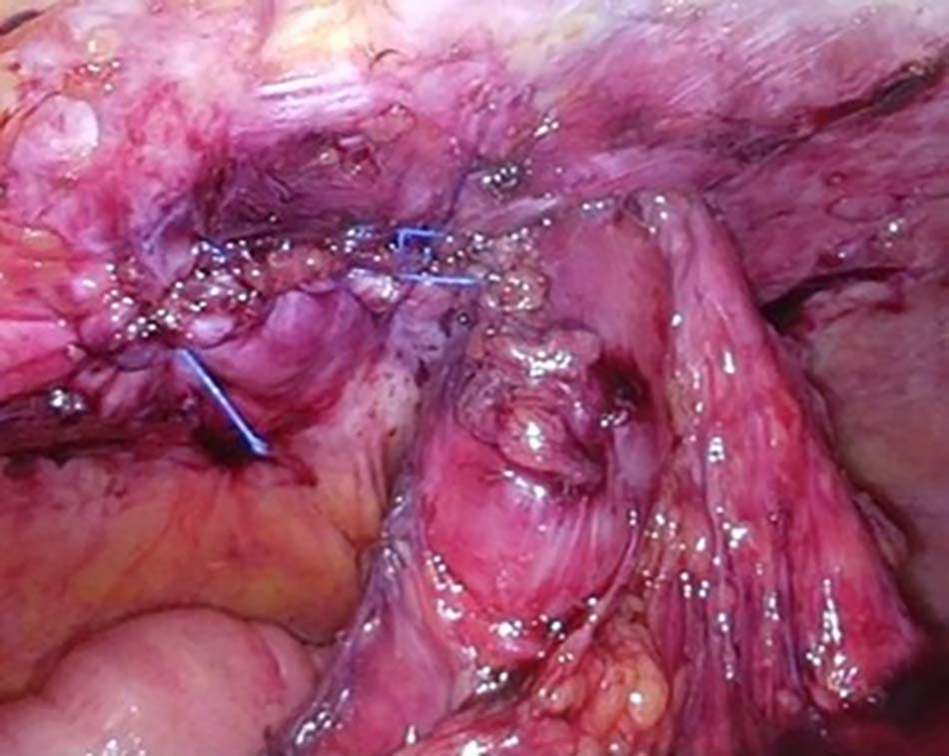
The hernia defect was closed with one Surgipro™ and 3-0Vloc™ suture.

**Figure 5:**
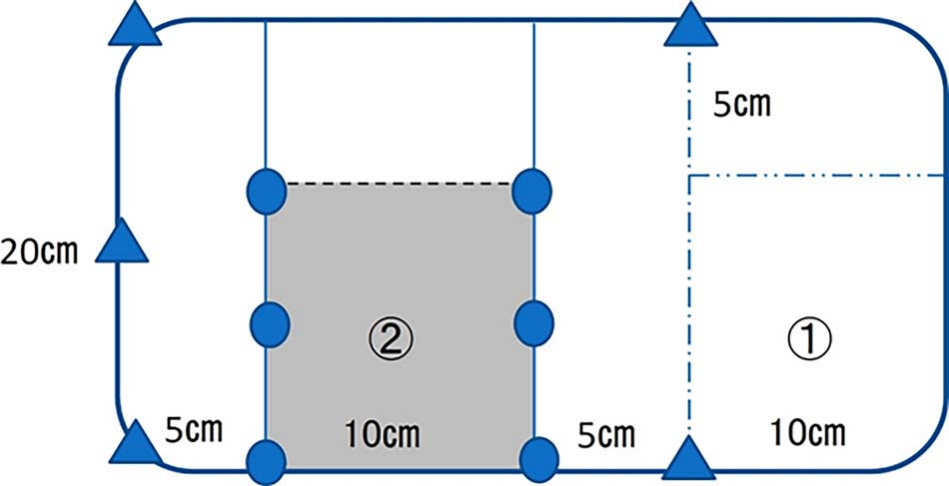
30 × 20 cm mesh was cut into 20 × 20 cm, and the remaining 10 × 10 cm part (①) was turned over and adjusted so that the collagen film (②); the corners of the mesh were fixed 3-0 nylon (triangle) for traction, and the folds in two with collagen film were fixed 3-0 VICRYL™ (circle).

**Figure 6:**
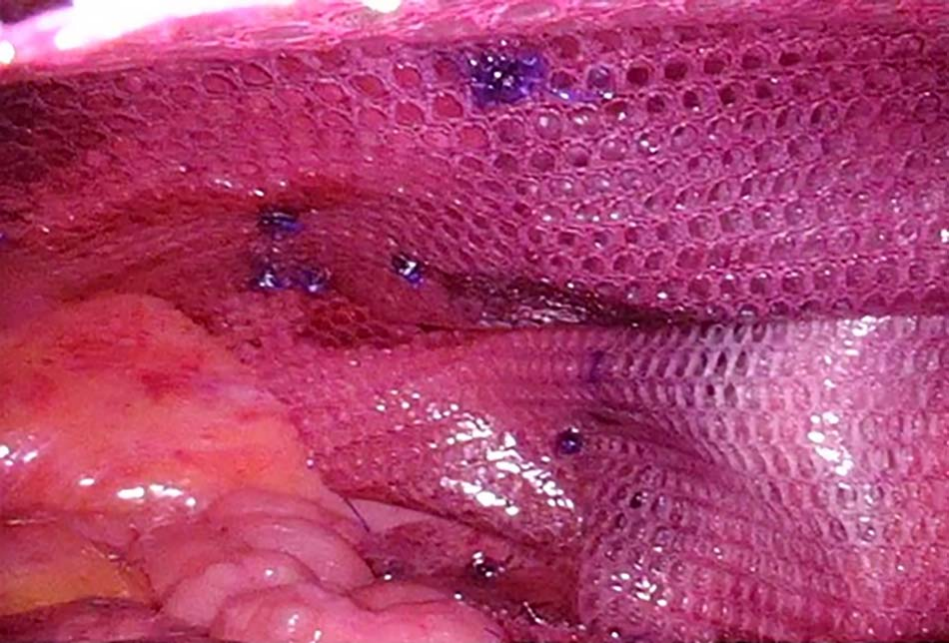
The mesh was fixed using Absorbable Tack™.

**Figure 7:**
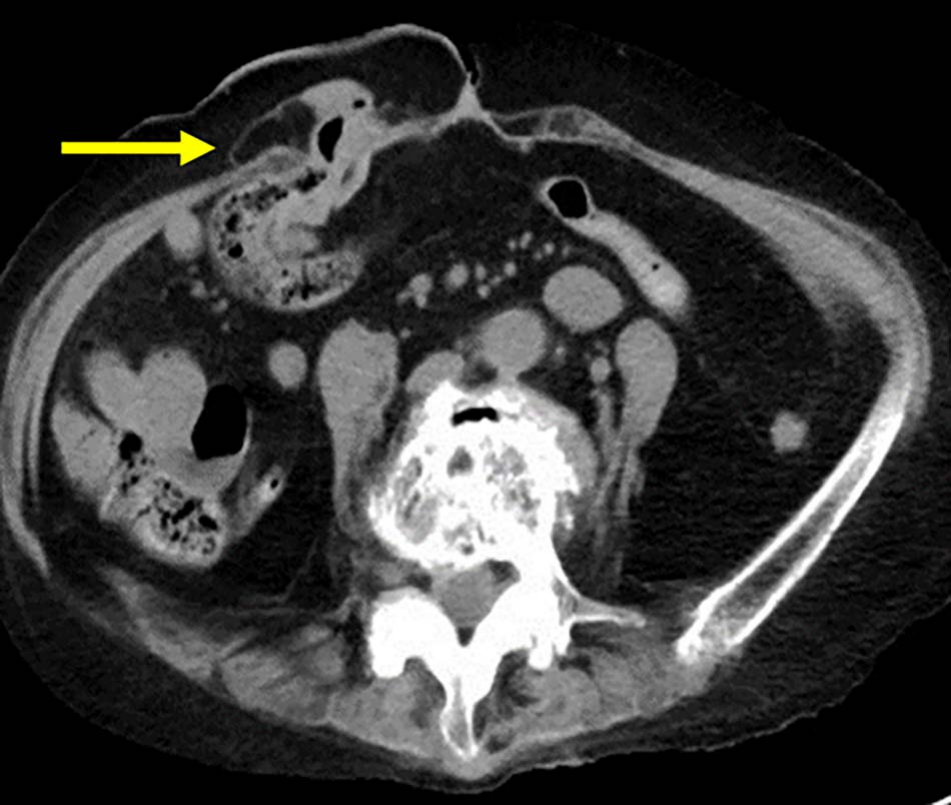
CT scan revealed no recurrence of the transverse colon on the oral side (arrow).

The patient was placed in the supine position. Three ports were used: two 5-mm ports and a 12-mm port placed vertically in the left abdominal (Fig. 2). Adhesions were found around the stoma, and the hernial orifice was exposed while paying attention to damage to the transverse colon and mesentery. Intestinal blood flow was evaluated using intraoperative indocyanine green fluorescent imaging to ensure no damage to the intestinal tract or mesentery (Fig. 3). The hernia defect was closed with one Surgipro™ and 3–0 Vloc™ suture (Fig. 4). The Symbotex Composite Mesh™ 30 × 20 cm was cut into 20 × 20-cm pieces, and the remaining 10 × 10 cm part was turned over and adjusted so that the collagen film touched the transverse colon. The corners of the mesh were fixed with 3-0 nylon for traction, and the folds with collagen film were fixed with 3-0 VICRYL™ (Fig. 5). The mesh was placed in the abdominal space and fixed using an absorbable Tack™ (Fig. 6). The patient was transferred to the Department of Internal Medicine on post-operative day 10 without any post-operative complications. There was no recurrence 6 months after surgery (Fig. 7).

## DISCUSSION

PSH is the most common complication of end colostomy and reduces the quality of life [5]. PSH repair results are unsatisfactory, as the reported recurrence rate after primary repair may be as high as 0–50%, with a high rate of surgery-related morbidity [6]. In addition, no specific recommendation for the optimal repair technique exists because of a lack of evidence [7]. The keyhole technique may lead to a recurrence rate of over 20% [8]; therefore, it should be avoided, as recommended by the EHS [4]. Suture repair and ostomy replacement have mainly been abandoned due to high recurrence rates [9]. The Sugarbaker technique, first described in 1985, is superior to the keyhole technique in both open and laparoscopic PSH repairs because of its lower recurrence rate and lack of increased risk of morbidity [10]. However, according to reports and case series publications, the sandwich technique may have better outcomes than the keyhole and Sugarbaker techniques [11]. The PSH repair results following both the Sugarbaker and sandwich techniques were reported to be significantly better than those following the keyhole technique [11]. There is also a method of converting the stoma into a retroperitoneum and recreating it; however, since the procedure becomes complicated, the Sugarbaker method using a mesh was adopted instead.

Parietex Composite Parastomal Mesh Sugarbaker™, which is often used, is subject to product collection because of the possibility of mesh breakage. In addition, the sandwich technique is not widely used owing to the complicated procedure and poor long-term results due to the overlapping mesh. In this study, we used the Symbotex Composite Mesh™ so that the collagen film surface touched both the transverse colon and the intra-abdominal organ side. By folding the mesh into two, the risk of breakage can be reduced, and intestinal injury can be prevented. On the other hand, mesh creation is complicated, and the thickness of the fold may make the fixing fragile. It was suggested that it could be dealt with by increasing the tacking and that it would be an effective technique.

In conclusion, we report excellent outcomes of the laparoscopic modified ‘Sugarbaker method’ using a collagen-coated Symbotex Composite Mesh™ to repair abdominal PSH. Since our case is a short-term result of 6 months, further follow-up is required; however, it is hoped that a better mesh for laparoscopic modified ‘Sugarbaker method’ will be developed in the future.
